# Supplementary material for: Lower Serum Uric Acid Levels May Lower the Incidence of Diabetic Chronic Complications in U.S. Adults Aged 40 and Over
Source: J Clin Med. 2023 Jan 16;12(2):725. doi: 10.3390/jcm12020725 (PMC9862742; doi:10.3390/jcm12020725)
Supplement: Supplementary file 1 [file jcm-12-00725-s001.zip › jcm-2062085-supplementary.pdf]

Supplementary Materials:

Table S1. Clinical features of the involved diabetes patients for the analysis of diabetic kidney disease (*n* = 3075).
Table S2. Clinical features of the involved diabetes patients for the analysis of diabetic peripheral neuropathy (*n* = 1453).
Table S3. Clinical features of the involved diabetes patients for the analysis of diabetic retinopathy (*n* = 1233).
Table S4. Clinical features of diabetic patients aged 40 and over with or without diabetic kidney disease (*n* = 3075).
Table S5. Clinical features of diabetic patients aged 40 and over with or without cardiovascular disease (*n* = 3106).
Table S6. Clinical features of diabetic patients aged 40 and over with or without diabetic peripheral neuropathy (*n* = 1453).
Table S7. Clinical features of diabetic patients aged 40 and over with or without diabetic retinopathy (*n* = 1233).

Table S1. Clinical features of the involved diabetes patients for the analysis of diabetic kidney disease (*n* = 3075).

SUA Levels	SUA≤300	300 <SUA≤ 420	SUA>420	*P* Value
Age (year) †	62 (19)	65 (16)	67 (16)	<0.01
Males (%) ‡	479 (42.9)	720 (53.3)	385 (63.2)	<0.01
Race (%) ‡				<0.01
Mexican American	365 (32.7)	320 (23.7)	59 (9.7)	
Other Hispanic	81 (7.3)	75 (5.6)	25 (4.1)	
Non-Hispanic White	407 (36.5)	583 (43.2)	281 (46.1)	
Non-Hispanic Black	221 (19.8)	334 (24.7)	219 (36.0)	
Other race	42 (3.8)	38 (2.8)	25 (4.1)	
Education level (%) ‡				<0.01
Less than 9th grade	325 (29.1)	356 (26.4)	107 (17.6)	
9–11th grade	237 (21.2)	235 (17.4)	128 (21.0)	
High school graduate	219 (19.6)	303 (22.4)	165 (27.1)	
College or AA degree	234 (21.0)	283 (21.0)	131 (21.5)	
College graduate or above	101 (9.1)	171 (12.7)	77 (12.6)	
Waist circumference (cm) †	101.8 (17.4)	107.7 (19.2)	110.3 (19.5)	<0.01
Cholesterol (mmol/L) †	5.04 (1.47)	4.94 (1.50)	4.86 (1.50)	0.083
Triglyceride (mmol/L) †	1.72 (1.36)	1.83 (1.57)	1.90 (1.50)	<0.01
Creatinine (μmol/L) †	70.7 (26.52)	79.6 (26.54)	99.0 (51.27)	<0.01
Poverty income ratio < 1 (%) ‡	234 (22.9)	279 (22.6)	99 (18.0)	0.051
Serum uric acid (μmol/L) †	255.8 (53.6)	350.9 (53.6)	469.9 (68.3)	<0.01
Glycohemoglobin (%) †	7.2 (2.4)	6.7 (1.5)	6.6 (1.3)	<0.01
Taking insulin now (%) ‡	203 (18.4)	220 (16.5)	130 (21.6)	0.059
Fasting glucose (mmol/L) †	7.88 (4.94)	7.22 (3.23)	7.16 (2.77)	<0.01
Duration of diabetes (year) †	9 (13)	8 (13)	10 (15)	0.034
Hypertension (%) ‡	588 (52.7)	904 (67.0)	476 (78.2)	<0.01
Smoked at least 100 cigarettes in life (%) ‡	559 (50.1)	724 (53.6)	361 (59.3)	<0.01

SUA: Serum uric acid. Data are number of subjects (percentage) or medians (interquartile ranges). † Kruskal–Wallis test was used to compare the median values among participants in different groups. ‡ Chi-square test was used to compare the percentage among participants in different groups.

Table S2. Clinical features of the involved diabetes patients for the analysis of diabetic peripheral neuropathy (n = 1453).

SUA Levels	SUA≤300	300 <SUA≤ 420	SUA>420	p Value
Age (year) †	62 (18)	65 (17)	67 (15)	<0.01
Males (%) ‡	260 (46.4)	371 (57.7)	157 (62.8)	<0.01
Race (%) ‡				<0.01
Mexican American	219 (39.1)	174 (27.1)	28 (11.2)	
Other Hispanic	26 (4.6)	26 (4.0)	10 (4.0)	
Non-Hispanic White	194 (34.6)	273 (42.5)	119 (47.6)	
Non-Hispanic Black	97 (17.3)	152 (23.6)	80 (32.0)	
Other race	24 (4.3)	18 (2.8)	13 (5.2)	
Education level (%) ‡				<0.01
Less than 9th grade	179 (32.0)	175 (27.2)	48 (19.2)	
9–11th grade	118 (21.1)	110 (17.1)	54 (21.6)	
High school graduate	96 (17.1)	140 (21.8)	71 (28.4)	
College or AA degree	111 (19.8)	137 (21.3)	51 (20.4)	
College graduate or above	56 (10.0)	81 (12.6)	26 (10.4)	
Waist circumference (cm) †	101.0 (16.2)	106.7 (18.6)	109.0 (20.5)	<0.01
Cholesterol (mmol/L) †	5.09 (1.40)	5.07 (1.50)	5.09 (1.36)	0.774
Triglyceride (mmol/L) †	1.67 (1.29)	1.78 (1.54)	1.96 (1.46)	<0.01
Creatinine (μmol/L) †	70.7 (26.52)	79.6 (26.54)	97.2 (53.04)	<0.01
Poverty income ratio < 1 (%) ‡	118 (22.9)	135 (23.1)	37 (16.4)	0.090
Serum uric acid (μmol/L) †	255.8 (59.5)	350.9 (53.6)	469.9 (59.4)	<0.01
Glycohemoglobin (%) †	7.6 (2.5)	6.7 (1.5)	6.7 (1.4)	<0.01
Taking insulin now (%) ‡	107 (19.5)	110 (17.4)	47 (19.3)	0.632
Fasting glucose (mmol/L) †	7.77 (5.11)	7.11 (3.22)	6.94 (2.75)	<0.01
Duration of diabetes (year) †	9 (16)	7 (14)	9 (17)	0.054
Hypertension (%) ‡	291 (52.0)	444 (69.2)	190 (76.0)	<0.01
Smoked at least 100 cigarettes in life (%) ‡	289 (51.6)	362 (56.3)	153 (61.2)	<0.01

SUA: Serum uric acid. Data are number of subjects (percentage) or medians (interquartile ranges). † Kruskal–Wallis test was used to compare the median values among participants in different groups. ‡ Chi-square test was used to compare the percentage among participants in different groups.

Table S3. Clinical features of the involved diabetes patients for the analysis of diabetic retinopathy (n = 1233).

SUA Levels	SUA≤300	300 <SUA≤ 420	SUA>420	p Value
Age (year) †	61 (18)	64 (17)	65 (17)	<0.01
Males (%) ‡	168 (39.7)	275 (50.5)	174 (65.7)	<0.01
Race (%) ‡				<0.01
Mexican American	102 (24.1)	114 (20.9)	21(7.9)	
Other Hispanic	42 (9.9)	38 (7.0)	14 (5.3)	
Non-Hispanic White	170 (40.2)	242 (44.4)	119 (44.9)	
Non-Hispanic Black	95 (22.5)	138 (25.3)	107 (40.4)	
Other race	14 (3.3)	13 (2.4)	4 (1.5)	
Education level (%) ‡				0.017
Less than 9th grade	86 (20.3)	126 (23.1)	38 (14.3)	
9–11th grade	90 (21.3)	94 (17.2)	51 (19.2)	
High school graduate	108 (25.5)	131 (24.0)	72 (27.2)	
College or AA degree	102 (24.1)	114 (20.9)	67 (25.3)	
College graduate or above	37 (8.7)	80 (14.7)	37 (14.0)	
Waist circumference (cm) †	102.4 (18.7)	108.7 (19.8)	112.2 (19.9)	<0.01
Cholesterol (mmol/L) †	4.91 (1.60)	4.78 (1.44)	4.66 (1.59)	0.152
Triglyceride (mmol/L) †	1.73 (1.38)	1.87 (1.53)	1.89 (1.54)	0.045

Creatinine (μmol/L) †	70.7 (25.33)	81.3 (26.52)	99.0 (42.88)	<0.01
Poverty income ratio < 1 (%) ‡	80 (20.6)	98 (19.4)	44 (18.1)	0.736
Serum uric acid (μmol/L) †	255.8 (53.6)	350.9 (53.6)	463.9 (71.3)	<0.01
Glycohemoglobin (%) †	6.9 (2.3)	6.6 (1.6)	6.5 (1.2)	<0.01
Taking insulin now (%) ‡	69 (16.3)	76 (13.9)	59 (22.3)	0.011
Fasting glucose (mmol/L) †	7.94 (4.61)	7.27 (3.16)	7.38 (2.60)	<0.01
Duration of diabetes (year) †	8 (11)	7 (10)	10 (12)	0.174
Hypertension (%) ‡	229 (54.1)	360 (66.1)	212 (80.0)	<0.01
Smoked at least 100 cigarettes in life (%) ‡	216 (51.1)	296 (54.3)	158 (59.6)	0.193

SUA: Serum uric acid. Data are number of subjects (percentage) or medians (interquartile ranges). † Kruskal–Wallis test was used to compare the median values among participants in different groups. ‡ Chi-square test was used to compare the percentage among participants in different groups.

Table S4. Clinical features of diabetic patients aged 40 and over with or without diabetic kidney disease ($n = 3075$).

Sua Levels	Diabetic Kidney Disease	Non-Diabetic Kidney Disease	p Value
Age (year) †	69 (16)	62 (17)	<0.01
Males (%) ‡	737 (52.7)	847 (50.5)	0.23
Race (%) ‡			0.53
Mexican American	328 (23.5)	416 (24.8)	
Other Hispanic	77 (5.5)	104 (6.2)	
Non-Hispanic White	599 (42.8)	672 (40.1)	
Non-Hispanic Black	350 (25.0)	424 (25.3)	
Other race	44 (3.1)	61 (3.6)	
Education level (%) ‡			<0.01
Less than 9th grade	400 (28.6)	388 (23.1)	
9–11th grade	299 (21.4)	301 (17.9)	
High school graduate	296 (21.2)	391 (23.3)	
College or AA degree	273 (19.5)	375 (22.4)	
College graduate or above	129 (9.2)	220 (13.1)	
Waist circumference (cm) †	105.7 (19.4)	106.2 (19.2)	0.740
Cholesterol (mmol/L) †	4.94 (1.63)	4.99 (1.40)	0.618
Triglyceride (mmol/L) †	1.86 (1.55)	1.75 (1.42)	0.022
Creatinine (μmol/L) †	96.4 (53.0)	70.7 (26.5)	<0.01
Poverty income ratio < 1 (%) ‡	289 (22.9)	323 (20.9)	0.215
Serum uric acid (μmol/L) †	350.9 (136.8)	315.2 (113.0)	<0.01
Glycohemoglobin (%) †	6.9 (1.9)	6.7 (1.6)	<0.01
Taking insulin now (%) ‡	344 (24.8)	209 (12.6)	<0.01
Fasting glucose (mmol/L) †	7.55 (4.16)	7.27 (3.31)	<0.01
Duration of diabetes (year) †	11 (15)	6 (10)	<0.01
Hypertension (%) ‡	996 (71.3)	972 (58.0)	<0.01
Smoked at least 100 cigarettes in life (%) ‡	767 (54.9)	877 (52.3)	0.354

Data are number of subjects (percentage) or medians (interquartile ranges). † Kruskal–Wallis test was used to compare the median values among participants in different groups. ‡ Chi-square test was used to compare the percentage among participants in different groups.

Table S5. Clinical features of diabetic patients aged 40 and over with or without cardiovascular disease ($n = 3106$).

SUA Levels	Cardiovascular Disease	Non-Cardiovascular Disease	p Value
Age (year) †	69 (15)	63 (18)	<0.01
Males (%) ‡	506 (56.9)	1099 (49.6)	<0.01
Race (%) ‡			<0.01
Mexican American	155 (17.4)	593 (23.8)	
Other Hispanic	33 (3.7)	150 (6.8)	
Non-Hispanic White	442 (49.7)	840 (37.9)	
Non-Hispanic Black	230 (25.9)	556 (25.1)	
Other race	29 (3.3)	78 (3.5)	
Education level (%) ‡			0.11
Less than 9th grade	218 (24.5)	580 (26.2)	
9–11th grade	196 (22.0)	412 (18.6)	
High school graduate	201 (22.6)	490 (22.1)	
College or AA degree	186 (20.9)	467 (21.1)	
College graduate or above	87 (9.8)	267 (12.0)	
Waist circumference (cm) †	107.2 (18.0)	105.2 (19.6)	<0.01
Cholesterol (mmol/L) †	4.68 (1.53)	5.07 (1.47)	<0.01
Triglyceride (mmol/L) †	1.84 (1.52)	1.78 (1.45)	0.81
Creatinine (μmol/L) †	88.4 (44.2)	76.0 (28.3)	<0.01
Poverty income ratio < 1 (%) ‡	175 (21.7)	436 (21.5)	0.96
Serum uric acid (μmol/L) †	350.9 (139.8)	321.2 (118.9)	<0.01
Glycohemoglobin (%) †	6.8 (1.6)	6.8 (1.9)	0.079
Taking insulin now (%) ‡	236 (26.8)	323 (14.7)	<0.01
Fasting glucose (mmol/L) †	7.27 (3.66)	7.44 (3.66)	0.105
Duration of diabetes (year) †	11 (16)	8 (12)	<0.01
Hypertension (%) ‡	696 (78.3)	1298 (58.6)	<0.01
Smoked at least 100 cigarettes in life (%) ‡	525 (59.1)	1142 (51.5)	<0.01

Data are number of subjects (percentage) or medians (interquartile ranges). † Kruskal–Wallis test was used to compare the median values among participants in different groups. ‡ Chi-square test was used to compare the percentage among participants in different groups.

Table S6. Clinical features of diabetic patients aged 40 and over with or without diabetic peripheral neuropathy ($n = 1453$).

SUA Levels	Peripheral Neuropathy	Non-Peripheral Neuropathy	p Value
Age (year) †	68 (16)	64 (17)	<0.01
Males (%) ‡	260 (64.5)	528 (50.3)	<0.01
Race (%) ‡			0.12
Mexican American	110 (27.3)	311 (29.6)	
Other Hispanic	10 (2.5)	52 (5.0)	
Non-Hispanic White	179 (44.4)	407 (38.8)	
Non-Hispanic Black	88 (21.8)	241 (23.0)	
Other race	16 (4.0)	39 (3.7)	
Education level (%) ‡			0.18
Less than 9th grade	124 (30.8)	278 (26.5)	
9–11th grade	76 (18.9)	206 (19.6)	
High school graduate	91 (22.6)	216 (20.6)	
College or AA degree	68 (16.9)	231 (22.0)	
College graduate or above	44 (10.9)	119 (11.3)	
Waist circumference (cm) †	106.7 (19.4)	104.5 (17.8)	0.013
Cholesterol (mmol/L) †	4.91 (1.40)	5.12 (1.43)	<0.01
Triglyceride (mmol/L) †	1.77 (1.24)	1.78 (1.51)	0.241

Creatinine (μmol/L) †	88.4 (35.4)	70.7 (26.5)	<0.01
Poverty income ratio < 1 (%) ‡	77 (20.7)	213 (22.3)	0.52
Serum uric acid (μmol/L) †	339.0 (130.9)	321.2 (118.9)	0.021
Glycohemoglobin (%) †	7.1 (2.1)	6.9 (1.9)	0.229
Taking insulin now (%) ‡	110 (27.7)	154 (15.0)	<0.01
Fasting glucose (mmol/L) †	7.60 (4.72)	7.22 (3.61)	0.016
Duration of diabetes (year) †	12 (18)	7 (12)	<0.01
Hypertension (%) ‡	277 (68.7)	648 (61.8)	0.014
Smoked at least 100 cigarettes in life (%) ‡	220 (54.6)	584 (55.6)	0.72

Data are number of subjects (percentage) or medians (interquartile ranges). † Kruskal–Wallis test was used to compare the median values among participants in different groups. ‡ Chi-square test was used to compare the percentage among participants in different groups.

Table S7. Clinical features of diabetic patients aged 40 and over with or without diabetic retinopathy (n = 1233).

SUA Levels	Diabetic Retinopathy	Non-Diabetic Retinopathy	*p* Value
Age (year) †	63 (15)	63 (18)	0.465
Males (%) ‡	234 (54.8)	383 (47.5)	0.015
Race (%) ‡			0.016
Mexican American	82 (19.2)	155 (19.2)	
Other Hispanic	31 (7.3)	63 (7.8)	
Non-Hispanic White	161 (37.7)	370 (45.9)	
Non-Hispanic Black	142 (33.3)	198 (24.6)	
Other race	11 (2.6)	20 (2.5)	
Education level (%) ‡			0.021
Less than 9th grade	100 (23.4)	150 (18.6)	
9–11th grade	90 (21.1)	145 (18.0)	
High school graduate	93 (21.8)	218 (27.0)	
College or AA degree	102 (23.9)	181 (22.5)	
College graduate or above	42 (9.8)	112 (13.9)	
Waist circumference (cm) †	107 (20.7)	107 (19.9)	0.508
Cholesterol (mmol/L) †	4.71 (1.68)	4.86 (1.47)	0.106
Triglyceride (mmol/L) †	1.71 (1.48)	1.85 (1.44)	0.022
Creatinine (μmol/L) †	81.3 (43.3)	79.6 (30.1)	0.026
Poverty income ratio < 1 (%) ‡	83 (21.5)	139 (18.5)	0.23
Serum uric acid (μmol/L) †	333.1 (124.9)	339 (124.9)	0.653
Glycohemoglobin (%) †	7.3 (2.3)	6.5 (1.2)	<0.01
Taking insulin now (%) ‡	143 (33.5)	61 (7.6)	<0.01
Fasting glucose (mmol/L) †	8.05 (5.22)	7.30 (2.61)	<0.01
Duration of diabetes (year) †	12 (12.3)	5 (8)	<0.01
Hypertension (%) ‡	296 (69.3)	505 (62.7)	0.02
Smoked at least 100 cigarettes in life (%) ‡	224 (52.5)	446 (55.3)	0.47

Data are number of subjects (percentage) or medians (interquartile ranges). † Kruskal–Wallis test was used to compare the median values among participants in different groups. ‡ Chi-square test was used to compare the percentage among participants in different groups.
